# Changes in hepatic L-carnitine levels in people with metabolic dysfunction-associated steatotic liver disease (MASLD) assessed by magnetic resonance techniques

**DOI:** 10.1038/s41598-026-54069-z

**Published:** 2026-05-22

**Authors:** Dragana Savic, Michael Pavlides, Mette Skalshøi Kjær, Sher May Ng, Katharine Thomas, Tim James, Gabrielle Allen, Claire Hart, Stefan Neubauer, Leanne Hodson, Ladislav Valkovič, Ferenc E. Mózes

**Affiliations:** 1 The Oxford Centre for Clinical Magnetic Resonance Research (OCMR), Radcliffe Department of Medicine, Level 0, John Radcliffe Hospital, University of Oxford, Headley Way, Oxford, OX3 9DU UK; 2 Oxford Centre for Diabetes, Endocrinology and Metabolism, University of Oxford, Oxford, UK; 3 Oxford NIHR Biomedical Research Centre, University of Oxford, Oxford, UK; 4 Translational Gastroenterology and Liver Unit, University of Oxford, Oxford, UK; 5 Novo Nordisk A/S, Copenhagen, Denmark; 6 Department of Clinical Biochemistry, Oxford University Hospitals NHS Foundation, Oxford, UK; 7 Department of Clinical Chemistry, Sheffield Children’s Hospital NHS Foundation, Sheffield, UK; 8 Department of Imaging Methods, Institute of Measurement Science, Slovak Academy of Sciences, Bratislava, Slovakia

**Keywords:** Biomarkers, Diseases, Gastroenterology, Medical research

## Abstract

L-carnitine is critical for metabolism of some fatty acids therefore different carnitine species may play a role in metabolic dysfunction-associated steatotic liver disease (MASLD) pathogenesis. Until recently, hepatic acetylcarnitine could not be quantified non-invasively. Now new technical developments in magnetic resonance spectroscopy (MRS) enable quantification of hepatic acetylcarnitine content in vivo. The aim of this study was to evaluate potential metabolic changes in healthy volunteers and patients with different stages of MASLD before and after a single injection of L-carnitine. This prospective study recruited 10 healthy volunteers and 17 patients (11 with low-risk MASLD, 6 with high-risk MASLD) who underwent cardiac and hepatic MRI and MRS for hepatic acetylcarnitine quantification at baseline and 2 h after injecting 50 mg/kg body weight L-carnitine. Serum samples were obtained for biomarker measurement. A single injection of L-carnitine significantly elevated hepatic acetylcarnitine levels in healthy volunteers (Δ_ACC_ = 0.172, *p* = 0.038) and low-risk MASLD (Δ_ACC_ = 1.677, *p* = 0.043), but not in high-risk MASLD (Δ_ACC_=-0.144, *p* = 0.397). However, high-risk MASLD showed elevated hepatic acetylcarnitine at baseline (Δ_ACC_ = 0.58, *p* = 0.007), whereas variability in low-risk MASLD masked differences vs. healthy volunteers. At baseline, serum medium-chain carnitine species were higher in the high-risk MASLD group (0.88 ± 0.28µmol/L, *p* = 0.002) and low-risk MASLD group (0.77 ± 0.49µmol/L, *p* = 0.03) compared to healthy volunteers (0.36 ± 0.23µmol/L). We showed for the first time that acetylcarnitine can be evaluated in vivo in patients with different stages of MASLD. The divergent hepatic responses to L-carnitine suggest stage-dependent differences in hepatic metabolic response that may reflect disease severity, potentially arising from differences in metabolic flexibility, carnitine transport, mitochondrial function, or baseline saturation of hepatic acetylcarnitine. Further studies are needed to confirm whether hepatic or circulating acetylcarnitine levels could serve as early biomarkers of MASLD progression.

**Supplementary Information:**

The online version contains supplementary material available at 10.1038/s41598-026-54069-z.

## Introduction

The global prevalence of metabolic dysfunction-associated steatotic liver disease (MASLD) is 25%^1^. Patients with MASLD have adverse metabolic profiles, with obesity, diabetes, hypertension, and dyslipidaemia often coexisting. As a result, 20–40% of patients with MASLD also have cardiovascular disease^2^. Patients with MASLD have disturbances in the metabolism of lipids and fatty acids (FA)^3^. Free L-carnitine is needed for the transportation of FAs into the mitochondria for beta-oxidation^4^, and it is also important for the modulation of intracellular acetyl-CoA/CoA ratio, thus controlling the balance between carbohydrate and FA oxidation (FAO) while additionally buffering excess acyl-CoA species to preserve mitochondrial CoA availability^5^.

Evidence is accumulating suggesting that L-carnitine may play a role in the pathogenesis of MASLD. Firstly, acylcarnitine species which are intermediates of FAO and made up of L-carnitine and a fatty acid chain of variable length (short, medium, and long chains of fatty acids) are known to be elevated in patients with MASLD and are associated with disease progression^6^. Furthermore, treatments with L-carnitine supplementation showed improvements in some aspects of MASLD^7^.

Efforts to understand the role of L-carnitine in health and disease have been hindered by the lack of reliable methods of in vivo quantification of carnitine species in tissues. Even though different carnitine species can be measured in serum, most L-carnitine is intracellular and therefore serum carnitine species poorly reflect whole-body and tissue carnitine^8^. Recent advances in ^1^H magnetic resonance spectroscopy (^1^H MRS) have enabled non-invasive tissue acetylcarnitine quantification using long echo time ^1^HMRS^9,10^. Several studies have optimized ^1^H MRS sequences for detecting acetylcarnitine in the muscle^9^, and a recent study has demonstrated the feasibility of measuring acetylcarnitine in the human liver^11^.

Beyond L-carnitine, hepatic lipid resonances such as allyl, methyl and methylene are also known to be elevated in patients with MASLD compared to healthy controls, as seen using ^1^H MRS^12^. In addition, all lipid resonances have a positive correlation with serum triglyceride levels, and Erickson et al. suggested that ^1^H MRS quantification of individual lipid resonances might have the potential to track the progression from MASLD to MASH^12^.

Having demonstrated the feasibility of acetylcarnitine quantification in the liver^11^, the aim of this study was to evaluate levels of L-carnitine and other metabolites in patients with MASLD and healthy volunteers at baseline and after intravenous supplementation with L-carnitine.

## Results

Baseline participant characteristics

Recruitment to the study started in December 2019 but had to be suspended in March 2020 due to the COVID19 pandemic. The study restarted again in May 2021 and recruitment was completed in April 2022. Ten healthy volunteers (mean age 32 years, 3 male) and 17 patients with MASLD (11 with low-risk MASLD and 6 with high-risk MASLD) were recruited. The baseline characteristics of the three groups are shown in Table 1 with additional details in Supplementary Table 1.

**Table 1:** Baseline characteristics of participants.

	Healthy (*n* = 10)	Low risk MASLD (*n* = 11)	High risk MASLD (*n* = 6)
Age (years)	32.5 ± 9.0	50.2 ± 17.2^**^	62.0 ± 5.8^****^
Sex (male %)	3 (33)	8 (73)	2 (33)
Weight (kg)	70.0 ± 16.4	98.9 ±18.4^**^	108.5 ±15.0^***^
BMI (kg/m^2^)	22.6 ± 2.1	33.5 ± 5.1^****^	40.2 ± 5.4^**** #^
Waist circumference (cm)	78 ± 10	107 ± 26^**^	123 ± 10^****^
Hip circumference (cm)	97 ± 6	109 ± 25	125 ± 13^***^
Waist-hip ratio	0.81 ± 0.12	0.99 ± 0.07^***^	0.99 ± 0.10^*^
Diabetes, n (%)	0	3 (27)	4 (66)
LSM-VCTE (kPa)	–	6.4 ± 1.5	20.7 ± 7.9^####^
CAP (dB/m)	–	318 ± 39	342 ± 53
PDFF (%)	1.9 ± 0.6	20.5 ± 10.8^****^	19.6 ± 10.8^***^

Data presented as mean ± standard deviation. Sex and diabetes status are presented as a number with a percentage from total participants in that group. Unpaired t-test and statistical significance is shown between MASLD and healthy volunteers as ^*^p = 0.05, ^**^p < 0.01, ^***^p < 0.001, ^****^ *p* < 0.0001. Statistical significance between low risk and high risk MASLD patients was encoded as ^#^*p* = 0.05, ^##^*p* < 0.01, ^###^*p* < 0.001, ^####^ *p* < 0.0001.

Abbreviations: Body mass index (BMI), liver stiffness measurement by vibration-controlled transient elastography (LSM VCTE), controlled attenuation parameter (CAP), proton-density fat fraction (PDFF).

### Baseline serum biochemistry

Alanine aminotransferase (ALT), aspartate aminotransferase (AST), gamma glutamyl transferase (GGT), and c-reactive protein (CRP) were significantly higher in the two MASLD groups compared to healthy volunteers. There was no significant difference between the two MASLD groups (Supplementary Table 2).

Fasting glucose (low-risk MASLD: 6.8 ± 2.4 mmol/L, high-risk MASLD: 8.1 ± 3.3 mmol/L) and ketone levels (low-risk MASLD: 0.46 mmol/L, high-risk MASLD: 0.33 mmol/L) were signficantly higher in the two MASLD groups compared to healthy volunteers (glucose: 4.9 ± 0.4 mmol/L, ketones: 0.1mmol/L; *p* < 0.05 for both). Insulin levels increased progressively from healthy volunteers (47 ± 17 pmol/L) to low-risk MASLD (119 ± 36 pmol/L; *p* < 0.05 compared to healthy volunteers) to high-risk MASLD (367 ± 348 pmol/L; *p* < 0.05 compared to low-risk MASLD). Homeostatic Model Assessment of Insulin Resistance (HOMA-IR) only reached significance in the low-risk MASLD (5.1 ± 2.1) compared to the healthy volunteers (1.5 ± 0.7, *p* < 0.0001). Low-risk MASLD had higher levels of triglycerides and apolipoprotein B (TAG: 2.8 ± 2.3 mmol/L, ApoB: 1.1 ± 0.3 g/L) compared to the healthy volunteers (TAG: 0.9 ± 0.4 mmol/L; ApoB: 0.8 ± 0.2 g/L; *p* < 0.05). There was no difference when comparing healthy volunteers to the high-risk MASLD group (TAG: 1.7 ± 1.2 mmol/L, ApoB: 0.8 ± 0.2 g/L). Cholesterol and ApoB was significantly lower in the high-risk MASLD group (3.8 ± 0.9 mmol/L) compared to the low-risk MASLD group (5.1 ± 1.4 mmol/L; *p* < 0.05), while there were no differences between healthy volunteers and the high-risk MASLD group. Lactate levels were similar between all groups (Supplementary Fig. 1). Several serum L-carnitine species were higher in the high-risk MASLD group compared to healthy volunteers (Supplementary Fig. 2).

### Baseline cardiac evaluation

Baseline cardiac function evaluation was notable for a higher heart rate (78 ± 21 bpm), left and right ventricular ejection fraction (LVEF: 67 ± 4%; RVEF: 68 ± 7%) and cardiac index (CI: 3.7 ± 1.1 L/min/m^2^) in patients with high-risk MASLD compared to healthy volunteers (HR: 53 ± 9 bpm, *p* = 0.007; LVEF: 62 ± 4%, *p* = 0.05; RVEF: 59 ± 4%, *p* = 0.006; CI: 2.7 ± 0.5 L/min/m^2^, *p* = 0.03) and there was no difference observed between high-risk MASLD compared to low-risk MASLD (HR: 67 ± 13 bpm, *p* = 0.19; LVEF: 63 ± 4.9%, *p* = 0.16; RVEF: 62 ± 7, *p* = 0.09; CI: 3.2 ± 0.7, *p* = 0.3). Furthermore, there was a stepwise increase in myocardial lipids as assessed by MRS from healthy volunteers (1.4 ± 0.5%) to low risk MASLD (1.66 ± 0.8%) to high risk MASLD (2.6 ± 2.1%). However, individual myocardial lipid resonances were significantly elevated in low-risk MASLD (allyl: 0.4 ± 0.2%, *p* = 0.02; methyl: 1.0 ± 0.5%, *p* = 0.0002; methylene: 1.7 ± 0.8%, *p* = 0.003) compared to healthy volunteers. N-terminal pro–B-type natriuretic peptide (NT-proBNP) from serum was within normal limits in low-risk MASLD (75 ± 83 pg/ml) and high-risk MASLD (73 ± 37 pg/ml). Details of the cardiac evaluations are shown in (Supplementary Table 3).

### Hepatic MRI/MRS at baseline

All individual lipid resonances from the liver were similar between the low- and high-risk MASLD groups but were all significantly higher in the two MASLD groups than in the healthy volunteers; individual peaks also correlated significantly with serum triglycerides (R^2^ = 0.2, *p* < 0.05). Saturation index was higher in the two MASLD groups compared to healthy volunteers, while unsaturation index was lower in the two MASLD groups compared to healthy volunteers (*p* < 0.001) (Supplementary Fig. 3).

Mean liver T_1_ was significantly longer in both MASLD groups compared to healthy volunteers (*p* < 0.05). Mean liver T_2_^*^ was similar across the three groups (Supplementary Table 4).

**Fig. 1:**
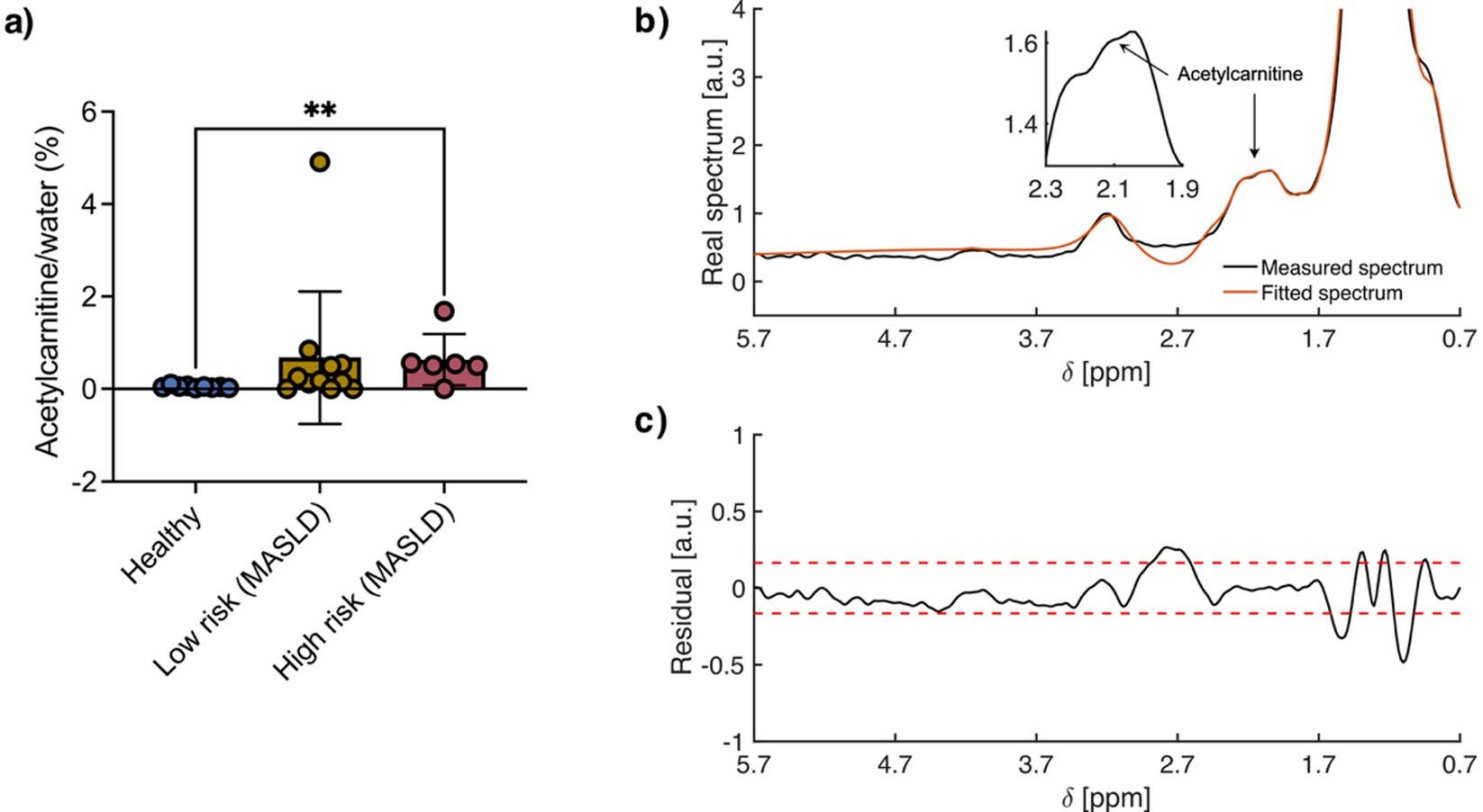
Baseline acetylcarnitine quantified in the liver using ^1^H MRS. (**a**) Acetylcarnitine levels normalized to unsuppressed water signal in healthy volunteers, low-risk MASLD and high-risk MASLD; (**b**) a representative measured and fitted spectrum showing the acetylcarnitine peak’s position (δ = 2.13 ppm) as evaluated in a voxel in the right liver lobe using a free-breathing STEAM sequence; (**c**) the residual corresponding to the fitting of the spectrum shown in panel b. Standardized unpaired t-test and statical significance is shown between healthy volunteers and high risk (MASLD) ^**^p = 0.007.

Acetylcarnitine levels measured with ^1^H MRS in the liver were significantly different in the high-risk MASLD group (0.63 ± 0.56%) compared to the healthy volunteers (0.05 ± 0.04%, *p* = 0.007), but no significant difference was detected between the low-risk MASLD (0.68 ± 1.4%, *p* = 0.18) and the healthy volunteers (Fig. 1).

### Changes from baseline following L-carnitine injection in MRI parameters of the liver and the heart

**Fig. 2:**
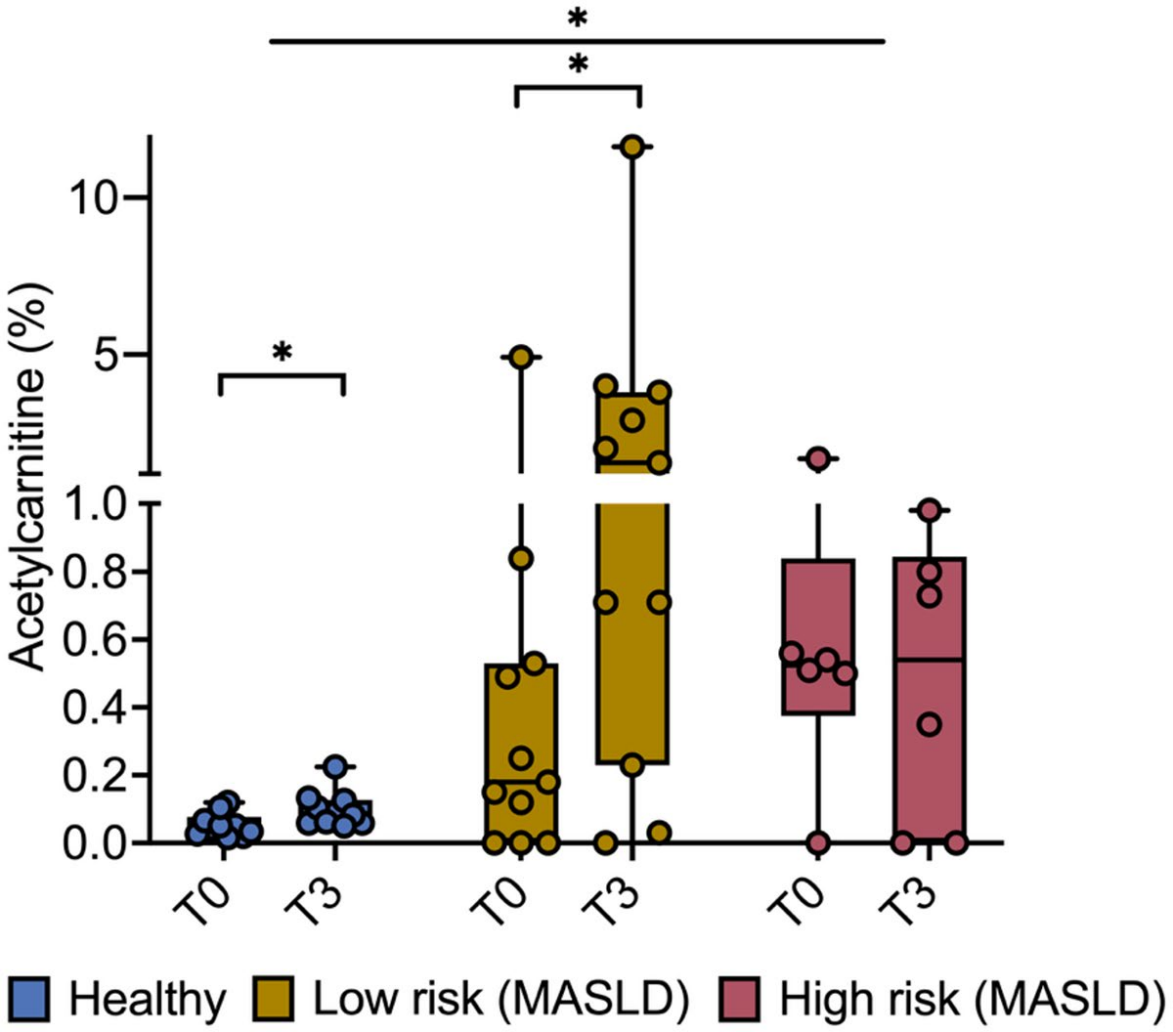
^1^H MRS of acetylcarnitine acquired from the liver. Acetylcarnitine/unsuppressed water. A two-way ANOVA analysis, where the three groups show statistical significance underlined with ^*^p < 0.05, and subsequent multiple comparisons between T0 and T3, where *p < 0.05.

There was a significant elevation of liver acetylcarnitine at time point T3 compared to baseline in healthy volunteers (86%, *p* = 0.04), and in patients with low-risk MASLD (820% change, *p* = 0.05), while the high-risk MASLD group showed no significant changes in acetylcarnitine (-25%, *p* = 0.6; Fig. 2). Two-way ANOVA analysis confirmed differences between the groups for all the liver parameters that were assessed without any effect from time point relative to carnitine injection and no interactions between time points and groups (Supplementary Table 5).

Inter-group differences were observed in left and right ejection fraction, cardiac index, as well as the allylic, methylene methyl resonances, and choline. There were no changes between group differences for total myocardial fat and there was no effect of the timepoint after injection of carnitine, or interactions between groups and timepoints (Supplementary Table 6).

### Changes from baseline following L-carnitine injection in serum metabolites

The proportional changes in serum metabolites between T0 and T3 sampling time points are shown in Supplementary Fig. 4, while all the time points were analysed using a two-way ANOVA which are shown in Supplementary Table 7. Patients with high-risk MASLD had greater relative change in glucose levels compared to healthy volunteers and had significantly greater relative change in TAG levels in the high-risk MASLD group compared to healthy volunteers (Supplementary Fig. 4).

There was a significant difference in the NEFA between the three groups (*p* = 0.032) but not with L-carnitine treatment (*p* = 0.39). Neither cholesterol nor HDL showed any difference between the groups or with L-carnitine treatment (Supplementary Table 7).

There was a significant increase in 3-OHB, AST, AST/ALT ratio, total bilirubin, and ApoB at time point T3 compared to baseline across all groups. At the same time, TAG, insulin, glucose, HOMA-IR urea, creatinine and lactate were lower at time point T3 compared to baseline (Supplementary Table 7).

**Fig. 3:**
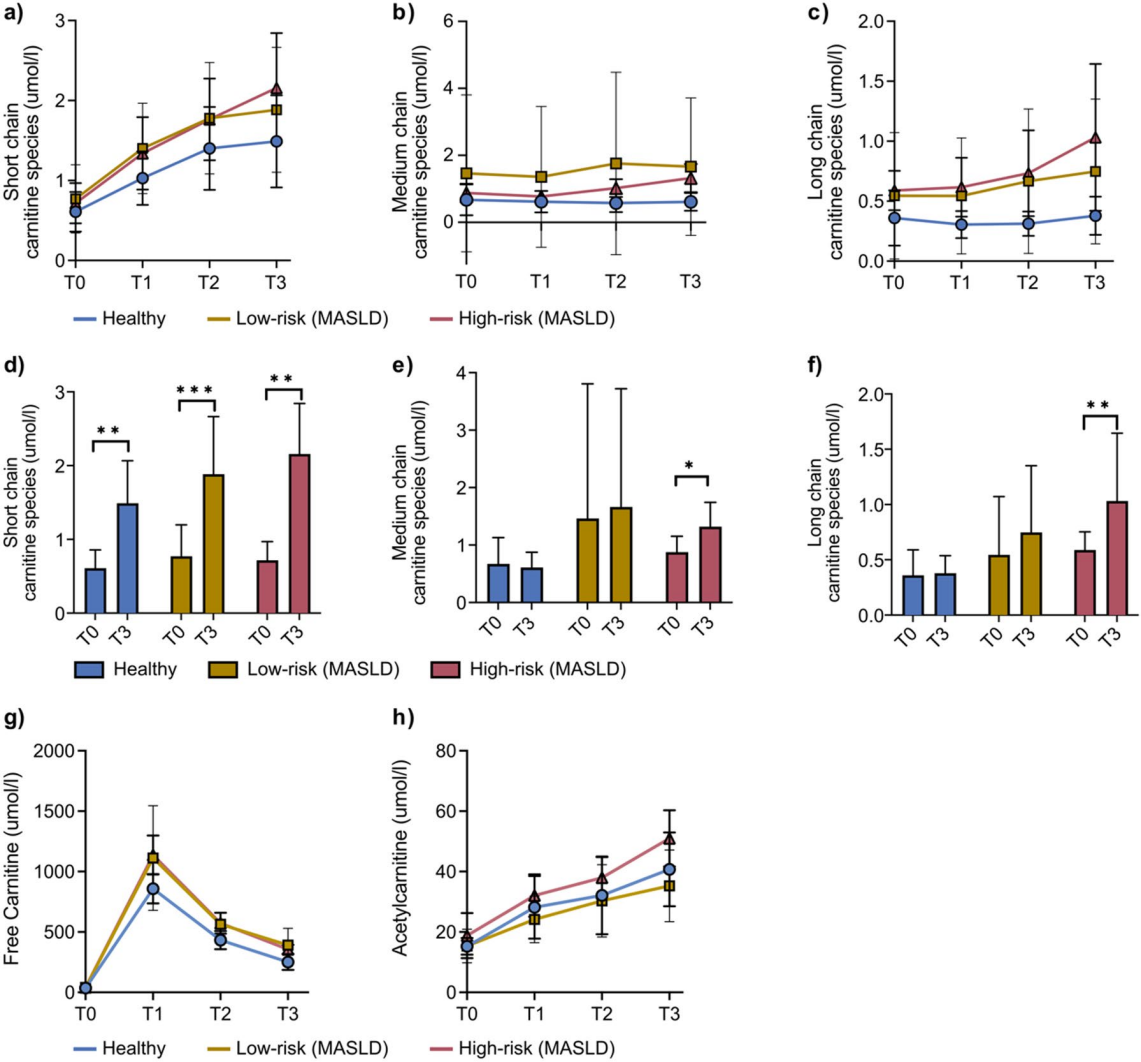
Serum carnitine species measured using tandem mass spectroscopy at baseline and at 1 h (T1), 2 h (T2) and 3 h (T3) after L-carnitine supplementation. Healthy volunteers (blue), low-risk MASLD (yellow), high-risk MASLD (red) (**a**) Short-chain carnitine species summed [C3-C5] (µmol/L), (**b**) medium-chain carnitine species summed [C6-C10] (µmol/L), (**c**) long-chain carnitine species summed [C12-C18] (µmol/L), (**d**) short-chain carnitine species summed [C3-C5] (µmol/L) at T0 and T3, (**e**) medium-chain carnitine species summed [C6-C10] (µmol/L) at T0 and T3, (**f**) long-chain carnitine species summed [C12-C18] (µmol/L) at T0 and T3. Serum free carnitine (**g**) and acetylcarnitine (**h**) levels are also shown at baseline and time points T1, T2, and T3. Two-way ANOVA was used with multiple comparisons to show any significant changes between T0 and T1, and between T0 and T3, where ^*^p < 0.05, ^**^p < 0.01, ^***^p < 0.001, ^****^p < 0.0001.

Short-chain carnitine species increased in all three groups between time points T0 and T3 (Fig. 3a, d). Medium-chain and long chain carnitine species increased at time point T3 compared to T0 only in high-risk MASLD (1.3 ± 0.4 µmol/L vs. 0.883 ± 0.3 µmol/L, Fig. 3b, e; and, respectively, 1.0 ± 0.6 µmol/L vs. 0.6 ± 0.2 µmol/L, Fig. 3c, f). As expected, there was an increase in serum free carnitine after the L-carnitine injection and a slow reduction during the later time points, while acetylcarnitine kept increasing over the following 3 h (Fig. 3g, h, Supplementary Table 8).

## Discussion

This study evaluated MRS and MRI imaging of the heart and the liver, as well as serum biomarkers before and after intravenous supplementation of L-carnitine in healthy volunteers, a group of low-risk and a group of high-risk MASLD patients. To our knowledge, this is the first time metabolic changes have been investigated in MASLD patients before and after an L-carnitine injection. Despite the acute supplementation, our study observed immediate changes in both carbohydrate and lipid metabolism. Furthermore, this study represents the first instance of measuring acetylcarnitine both in the blood and in the liver using in vivo MRS in the same patients. Hepatic acetylcarnitine levels in both healthy volunteers and low-risk MASLD patients increased after L-carnitine supplementation, but not in patients with high-risk MASLD, while these patients had higher baseline acetylcarnitine than participants in the other two groups.

Acetylcarnitine in the liver can be evaluated using in vivo MRS at a clinical field strength of 3 T, as it has recently been demonstrated^11^. Although stable hydrogen isotope fractionation (^2^H/^1^H) in fatty acids has been shown to act as a sensitive marker of metabolic routing and cellular redox state, reflecting large biosynthetic isotope effects linked to NADPH use^13^, this phenomenon does not influence conventional clinical measurements obtained with ^1^H MRS. At natural abundance, deuterium constitutes only a very small fraction of total hydrogen and is invisible to ^1^H MRS. Thus, even large biological shifts in lipid ^2^H/^1^H ratios do not measurably affect proton signal amplitudes of lipid or acetylcarnitine resonances and have not confounded our measurements.

This paper shows there is a difference between serum acetylcarnitine levels compared to hepatic acetylcarnitine levels in vivo, which might demonstrate earlier changes present in the liver. Previous studies have shown that serum levels of carnitine species are higher in MASLD and could be used for detecting disease progression^6^. This study supports those findings, demonstrating a higher numerical level of acetylcarnitine in both serum and hepatic levels in the high-risk MASLD group. Additionally, our study suggests that hepatic acetylcarnitine response to an L-carnitine challenge may capture earlier disease-related changes in MASLD, such as those present in low-risk MASLD patients, that are not fully reflected by serum acetylcarnitine response alone. Whilst serum acetylcarnitine increased in all groups following supplementation, the divergent hepatic response – with low-risk MASLD patients showing a marked hepatic increase and high-risk MASLD patients showing no significant hepatic increase – was not mirrored by differences in serum acetylcarnitine. These findings might indicate that detecting hepatic acetylcarnitine levels may provide more valuable information about the early stages of fatty liver disease, particularly when serum levels of acetylcarnitine fail to show new information. A previous study reported that serum acetylcarnitine levels in a cohort of MASLD patients were reduced and could be used as prognostic marker for hepatocellular carcinoma^14^. Our study showed no significant changes in circulating acetylcarnitine levels between volunteers and the two MASLD groups, but a significant elevation of liver-specific acetylcarnitine levels at baseline between healthy volunteers and the high-risk MASLD group. The lack of significant difference in liver acetylcarnitine between healthy volunteers and the low-risk MASLD group is likely attributable to the presence of outlier measurements. Serum acylcarnitine accumulation have also previously been suggested to be a surrogate marker for carnitine palmitoyl transferase (CPT2) downregulation, an enzyme that is critical for fatty acid oxidation and has been showed to have an active role in hepatocarcinogenesis^12^. Overall, these studies suggest that an imbalance in acetylcarnitine levels might suggest poor prognosis in patients with MASLD. Our study did not recruit any patients with hepatocellular carcinoma, however medium-chain carnitine species showed a significant elevation in the high-risk MASLD group compared to the healthy volunteers. Previous studies have shown that an accumulation of C6, C8 and C10 carnitine could also indicate that these patients are medium-chain acyl-coenzyme A dehydrogenase deficiency carriers^15^. Usually there is a prominent accumulation of serum medium-chain carnitine species in patients MASLD^6^, this is thought to occur due to impaired mitochondrial function in the liver, leading to incomplete beta-oxidation of fatty acids with subsequent accumulation of medium-chain acyl-CoA intermediates, which are converted to acylcarnitines and thereby released into the circulation. Indeed, in our study half of the patients with high-risk MASLD had such an accumulation (> 15% compared to clinical standard values), while 9 of the 11 patients with low-risk MASLD had a reduction of serum C8 species. In the early stages the liver still can effectively metabolize fatty acids, and therefore there might not be a build-up of acylcarnitine species in the bloodstream.

It should be noted that hepatic acetylcarnitine measurements were not available for 4 patients at baseline, and 3 patients at T3 time point, due to unquantifiable acetylcarnitine resonances. Given the small group size, and the fact that the divergent hepatic response is central to the main conclusions of this study, our findings should be interpreted with caution and require replication in larger cohorts.

Our study demonstrated that a single injection of L-carnitine led to changes in serum short-chain and long-chain carnitine species, while medium-chain carnitine species remained mostly unaffected. In both healthy volunteers and MASLD groups, serum acetylcarnitine levels increased following the L-carnitine injection, but there was no corresponding increase in hepatic acetylcarnitine levels in the high-risk MASLD group. This blunted hepatic response may reflect reduced metabolic flexibility, for example impaired handling of acetyl-CoA and its conversion to acetylcarnitine. Alternatively, differences in carnitine transport, mitochondrial function, or baseline saturation of hepatic acetylcarnitine could also contribute. Further mechanistic studies are needed to distinguish between these possibilities. Conversely, healthy volunteers and the low-risk MASLD group may have had a surplus of acetyl-CoA, allowing more of it to bind L-carnitine and form acetylcarnitine in the liver. This acetylcarnitine could then be transported into the mitochondria for beta-oxidation or serve as a buffer the excess acetyl-CoA generated from pyruvate dehydrogenase activity or beta-oxidation elsewhere (i.e. the systemic circulation)^5^. Further studies are needed to evaluate whether this mechanism of reduced or no capacity for metabolic liver flexibility is blunted in patients with high-risk MASLD. Using MRS as a tool to detect early changes in carnitine species may prove valuable for disease monitoring. Our findings suggest that serum- and tissue-specific markers do not reflect the same metabolic processes, and that hepatic MRS may capture earlier changes in acetylcarnitine concentration compared to serum markers. Further studies are needed to be able to understand the pool exchange between serum and tissue-specific levels of carnitine.

Baseline differences in age, BMI, and sex distribution between groups may also contribute to the observed differences in hepatic acetylcarnitine levels and response to L-carnitine. Notably, the high-risk MASLD group was significantly older and had a substantially higher BMI compared to healthy volunteers. Both age and obesity are associated with reduced mitochondrial function and altered carnitine metabolism independently of MASLD severity^16,17^. Whilst the primary aim of this exploratory study was to characterise differences between groups as defined, future studies with larger, better-matched cohorts or with covariate adjustment will be needed to disentangle disease-specific effects from those related to age, adiposity, and sex.

Several studies have shown the ability of L-carnitine to elevate pyruvate dehydrogenase flux and thereby reduce glucose levels and improve insulin levels^5,7,18^. We observed that HOMA-IR, glucose, and insulin levels were lower at T3 compared to baseline following a single injection of L-carnitine. However, as the study lacked a placebo-controlled arm, a causal relationship cannot be established, and these changes may partly reflect normal physiological variation over the duration of the study. Malaguarnera et al. 7. conducted a randomized controlled clinical trial investigating the effects of 24 weeks of oral treatment with L-carnitine in patients with high-risk MASLD in comparison with a placebo treatment group of equal length, showing that L-carnitine was effective in reducing total cholesterol and triglycerides, and improved insulin resistance^7^. Our study showed that just a single injection of L-carnitine was associated with lower glucose, insulin, and HOMA-IR levels, although cholesterol did not change. Cholesterol promotes the activation of Kupffer and stellate cells which induce inflammation and fibrosis^19^, therefore hepatic accumulation of cholesterol rather than triglycerides is associated with continued hepatocellular apoptosis and liver damage and might play a role in the prognosis of MASLD. We observed differences in triglycerides and other markers, such as 3-OHB, AST, urea, creatinine, total bilirubin, and ApoB in all three participant groups before and after L-carnitine supplementation. These changes suggest a fast action of L-carnitine to improve metabolic markers in vivo, and possibly the benefit of intravenous injections compared to oral treatments.

We also evaluated individual lipid resonances using MRS in both the heart and the liver. In line with earlier findings^12^, we found that patients with MASLD had higher allyl, methyl, and methylene concentrations and overall, more lipids were stored in the liver as saturated fat which has been suggested to induce lipotoxicity^20^ and less as unsaturated fat. No difference in liver fat composition was found between the two groups of patients with MASLD. Our study did not show any changes in the lipid resonances after an L-carnitine injection. However, other studies have shown L-carnitine treatment to be effective in reducing lipids when treated for a longer period of time^21,22^, although optimal dosages remain unclear for humans. Furthermore, lipid metabolism is a dynamic process, that is influenced by several factors, such as nutrient availability, hormonal signalling, and circadian rhythm. Therefore, the timing of L-carnitine injection may be just as important as the duration or dosage of L-carnitine treatment in impacting hepatic lipid metabolism. Based on current literature, we can only speculate that longer treatment with L-carnitine and carefully timed injection could lead to reductions of hepatic lipid peaks.

Despite no liver fat changes were observed using MRI, it is known that liver fat fraction decreases with increased severity of MASLD^23^, hence the secreted triglycerides in patients with high-risk MASLD are lower than in patients with low-risk MASLD. However, we did not observe a similar pattern on MRS of the lipid resonances. In addition, it has been shown that the synthetic function of the liver decreases with the severity of MASLD, and this was reflected in our cohort of patients by reduction in serum ApoB^24^.

In recent years there has been an increased focus on studying the relationship between the liver and the heart, known as the liver-heart axis^25^. Research has shown that patients with MASLD have double the risk of developing cardiovascular disease compared to patients without MASLD^26^. Our study used cardiac MRI for volumetric assessment and found that patients in the high-risk MASLD group had a higher left and right ventricular ejection fraction, and a higher cardiac index when compared to healthy volunteers, consistent with prior findings^27^. However, it is important to note that this difference may have been influenced by the advanced age of the patients in the high-risk MASLD group compared to healthy volunteers^28^. While our study showed that both the low-risk and high-risk MASLD groups were significantly older than the healthy volunteers, only the high-risk group exhibited higher ejection fraction and cardiac index. Therefore, it is possible that the higher ejection fraction and cardiac index observed in the high-risk MASLD group were influenced by both advanced age and the presence of MASLD. However, previous research has also shown a strong association between MASLD and increased risk of cardiovascular disease, suggesting that MASLD itself may also be a contributing factor^24,25^.

The presence of a hyperdynamic circulation in the MASLD group can be explained by a reduced systemic vascular resistance through splanchnic arterial vasodilation^29^. In addition, a reduced end-diastolic volume due to reduced venous filling from increased hepatic venous pressure further increases stroke volume, contributing to an increased cardiac index^30^. Even though we could not detect any functional differences in the low-risk MASLD group compared to the healthy volunteers, we showed early elevation of lipid resonant peaks in the cardiac septum, which were not present in the high-risk MASLD group. This might suggest that there are early cardiac metabolic changes, prior to the functional changes, as previously suggested^5^. Cardiac lipids were not different between the healthy volunteers and the high-risk MASLD group, where a functional difference was detected.

In conclusion, we demonstrated an association between MASLD disease severity and hepatic acetylcarnitine levels both at baseline and after an intravenous injection of L-carnitine. Our findings suggest that hepatic acetylcarnitine response to an intravenous L-carnitine supplementation may serve as a more sensitive functional marker of MASLD severity compared to serum acetylcarnitine response, capturing divergent metabolic behaviour between disease stages that is not apparent from serum measurements alone. Additionally, our study demonstrates the potential of cardiac and hepatic MRS in detecting early metabolic changes in MASLD patients. Further investigation is needed to explore the clearance of acylcarnitine species following L-carnitine supplementation, and longitudinal studies monitoring the progression of MASLD may benefit from early detection of hepatic acetylcarnitine levels.

## Methods

### Study design and subjects

Patients were recruited from hepatology clinics at the John Radcliffe Hospital (Oxford, UK). Patients had a liver stiffness measurement (LSM) and controlled attenuation parameter (CAP) measured by vibration-controlled transient elastography (VCTE). MASLD disease severity was classed as “low risk” if liver stiffness measurement by vibration controlled transient elastography (LSM-VCTE) was < 10 kPa, “intermediate risk” if LSM-VCTE was ≥ 10 kPa without evidence of cirrhosis, and “high risk” if LSM-VCTE was > 10 kPa with evidence of cirrhosis. Histological, clinical, and radiological diagnosis of cirrhosis was acceptable. Due to small numbers, patients with intermediate and high risk were grouped together and labelled as “high risk” group. Healthy volunteers were recruited via poster advertisements and word of mouth. Volunteers were eligible if they had no self-reported history of liver disease or other chronic health condition, consumed alcohol within the recommended limits (< 14 units/week irrespective of sex) and had BMI ≤ 25 kg/m^2^. The study was approved by the National Research Ethics Committee Service (Reference: 19/SC/0571) and was conducted in accordance with the 1975 Declaration of Helsinki. Written informed consent was obtained from all participants.

**Fig. 4:**
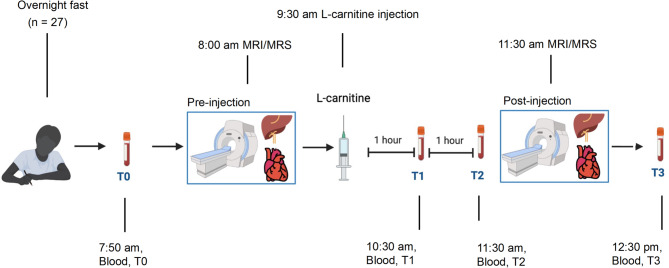
Diagram of experimental protocol. All participants attended in the morning following an overnight fast. Livers and hearts of 27 volunteers (10 healthy, 17 patients) were imaged in the morning and again 2 h later for a follow-up scan. All volunteers received a single injection of L-carnitine, and blood samples (at time points T0, T1, T2 and T3) were collected.

Study participants attended a single study visit at the Oxford Centre for Clinical Magnetic Resonance Research (Oxford, UK) after an overnight fast. No other instructions were given regarding following any specific diet in the days preceding the study visit. Participants underwent baseline MR assessments followed by an intravenous injection of L-carnitine (50 mg/kg body weight, Carnitor, Alfasigma S.p.A., Italy). MR assessments were repeated two hours after the L-carnitine injection. Blood samples were taken at four time points during the study (Fig. 4).

### MR data acquisition

All measurements were undertaken in subjects lying supine in a clinical 3T MR system (Prisma, Siemens Healthineers, Erlangen, Germany) using a 30-channel phased array coil and a 12-channel spine coil. The body coil of the scanner was used for excitation, and coils covered both the liver and heart without the need to move between acquisitions.

### Liver MRI

Liver MRI was used to quantify T1 and T2* relaxation times. Liver images were acquired in a single axial slice. T_1_ was measured using a standard shortened modified Look-Locker (shMOLLI)^31^ sequence. Three inversion pulses were followed by 5, 1 and 1 single shot balanced steady-state free precession (bSSFP) images synchronised to subjects’ electrocardiogram (ECG) (TR/TE = 2.43/1.05 ms, TI_min_ = 100 ms, TI_increment_ = 80 ms) with field of view (FoV) = 440 mm × 330 mm, slice thickness 8 mm, FA = 35°, and BW = 965 Hz/px. T2* values were quantified using a multiple-echo spoiled gradient-recalled echo sequence with 12 echo times, TR/TE_min_ = 15/1.1 ms, ΔTE = 1.1 ms, slice thickness 10 mm, FA = 3°, and bandwidth 1560 Hz/px^32^.

### Cardiac MRI

Cardiac volume and function were assessed using a cinematic bSSFP sequence (TR/TE = 3/1.5 ms, voxel size 2 × 2 × 8 mm^3^, FoV = 380 mm × 380 mm, FA = 55^◦^, matrix: 192 × 192, GRAPPA factor: 3, 24 reference lines, segments: 15, concatenations: 1), which was performed with cardiac triggering during end-expiratory breath-hold.

### Hepatic and cardiac MRS

Spectroscopic voxels were placed to avoid hepatic vasculature and bile ducts in the liver. Acetylcarnitine was quantified in the liver using a STEAM acquisition (64 averages, TR/TE = 3000/100 ms, voxel size: 30 × 30 × 30 mm^3^) allowing for free breathing.

Fat Fraction (FF) and lipid resonances^33^ were measured using a STEAM acquisition in the liver and in the cardiac septum. Three water-suppressed and one non-water-suppressed breath-held STEAM acquisitions were collected in the posterior part of the right liver lobe for lipid quantification. Five water-suppressed and one non-water-suppressed breath-held STEAM acquisitions were collected in the cardiac septum. The acquisitions were performed in breath-hold and were synchronised to subjects’ ECG trace (5 measurements for water-suppressed acquisitions and 3 measurements for non-water-suppressed acquisitions, TR/TE/TM = 760/10/10 ms, voxel size: 20 × 20 × 20 mm^3^) with a pause of 2 s between measurements for water-suppressed acquisitions, and with a pause of 4 s between measurements for non-water-suppressed acquisitions to minimize partial saturation. The timing of study visit elements is illustrated in Fig. 4.

### MR data analysis

Liver T2* maps were determined using the MAGO^34^ algorithm. T1 maps were reconstructed using the conditional fitting algorithm described by Piechnik et al.^31^. T1 and T2* were measured in a single region of interest on each slice and means were reported as described previously^33^.

Endocardial and epicardial left and right ventricular (LV and RV) contours were drawn manually and analysed using a semi-automated system (cmr42, Circle Cardiovascular Imaging Inc., Calgary, Canada). Cardiac MRI data were analysed by two experienced observers (SMN & KT) blinded to clinical data.

For acetylcarnitine quantitation hepatic spectra were frequency aligned using water as the reference resonance using the OXSA toolbox^35^ in Matlab (Mathworks, Natick, MA, USA). Frequency-aligned spectra were then phase corrected and averaged. The acetylcarnitine resonance at 2.1 ppm was fitted along with lipid resonances. For hepatic and cardiac lipid quantitation multiple resonances of fat were fitted; methyl (δ = 0.9 ppm), methylene (δ = 1.3 ppm), and allylic (δ = 2.2 ppm) peak were all quantified compared to the non-supressed water signal using Eq. (1).

1 $$\:Fat\:fraction=\:\frac{F\left(lipid\ peak\right)}{F\left(lipid\ peak\right)+W(non-supressed\ water\ peak)}\times\:100\%$$

### Blood samples

Blood samples were collected into serum SSTII tubes (Becton Dickinson Ltd, UK) centrifuged, and serum was immediately transferred to sterile polypropylene cryovials which were submerged in liquid nitrogen and transferred to a -80 °C freezer. Aliquots were sent to Clinical Biochemistry, Oxford University Hospitals NHS Foundation Trust for standard biomarker analysis. Standard laboratory methods were undertaken using the Abbott Architect i2000 (insulin and NT-proBNP) and the c16000 analyser (non-esterified fatty acids (NEFA), beta-hydroxybutyrate (BOHB), cholesterol, triglycerides (TAG), high-density lipoprotein (HDL), alanine aminotransferase (ALT), aspartate aminotransferase (AST), urea, creatine, C-reactive protein (CRP), total bilirubin (TBIL), glucose, gamma-glutamyl transferase (GGT), lactate, apolipoprotein B (ApoB)).

Free carnitine and the various acylcarnitine species were measured in the serum by an ISO 15189 (Medical Laboratory) accredited assay at the Department of Clinical Chemistry, Sheffield Children’s NHS Foundation Trust, where it is in routine clinical use for the investigation of individuals with potential congenital metabolic diseases. The method is a derivatised, stable isotope dilution LC-MS/MS method using a Waters TQD Tandem Mass Spectrometer with Waters 2777 C HPLC Auto-sampler.

The Homeostatic Model Assessment for Insulin Resistance (HOMA-IR) was calculated with Eq. (2).

2 $$\:\mathrm{H}\mathrm{O}\mathrm{M}\mathrm{A}-\mathrm{I}\mathrm{R}\:=\frac{Glucose(mmol/L)\times\:Insulin(mmol/L)}{22.5}$$

### Statistical methods

All data were presented as mean ± standard deviation (SD) unless otherwise stated. Baseline data were compared using a standardized t-test before any effects of L-carnitine were considered. Follow-up data was compared using a two-way ANOVA based on a generalized linear model (GLM) and with Šidák multiple comparisons correction using a 95% confidence interval. All statistical analysis was performed using Graphpad Prism 9 (San Diego, USA).

## Supplementary Information

Below is the link to the electronic supplementary material.

Supplementary Material 1

## Data Availability

The data that support the findings of this study are available from the corresponding author upon reasonable request.
