# Supplementary material for: Changes in hepatic L-carnitine levels in people with metabolic dysfunction-associated steatotic liver disease (MASLD) assessed by magnetic resonance techniques
Source: Sci Rep. 2026 May 22;16:23397. doi: 10.1038/s41598-026-54069-z (PMC13407869; doi:10.1038/s41598-026-54069-z)
Supplement: Supplementary file 1 — Supplementary Material 1 [file 41598_2026_54069_MOESM1_ESM.docx]

Supplementary material for “Changes in cardiac and hepatic L-carnitine levels in people with MASLD assessed by magnetic resonance techniques”

Dragana Savic^1,2*^, Michael Pavlides^1,3,4*^, Mette Skalshøi Kjær^5^, Sher May Ng^1^, Katharine Thomas^1^, Tim James^6^, Gabrielle Allen^6^, Claire Hart^7^, Stefan Neubauer^1^, Leanne Hodson^2,3^, Ladislav Valkovič^1,8^, Ferenc E. Mózes^1^

^1^The Oxford Centre for Clinical Magnetic Resonance Research (OCMR), Radcliffe Department of Medicine, University of Oxford, Oxford, UK

^2^Oxford Centre for Diabetes, Endocrinology and Metabolism, University of Oxford, Oxford, UK

^3^Oxford NIHR Biomedical Research Centre, University of Oxford, UK

^4^Translational Gastroenterology Unit, University of Oxford, Oxford, UK

^5^Novo Nordisk A/S, Denmark

^6^Department of Clinical Biochemistry, Oxford University Hospitals NHS Foundation Trust

^7^Department of Clinical Chemistry, Sheffield Children’s Hospital NHS Foundation Trust

^8^Department of Imaging Methods, Institute of Measurement Science, Slovak Academy of Sciences, Bratislava, Slovakia

*Contributed equally to this work

# Supplementary Tables

**Supplementary Table 1** Clinical details and severity group assignment for patients with non-alcoholic fatty liver disease

| **Sex** | **Age (yrs)** | **BMI (kg/m^2^)** | **DM** | **metformin** | **insulin** | **gliclazide** | **LS (kPa)** | **CAP (dB/m)** | **Biopsy** | **F**  **(0-4)** | **S**  **(0-3)** | **LI**  **(0-3)** | **B**  **(0-2)** | **Severity group** |
| --- | --- | --- | --- | --- | --- | --- | --- | --- | --- | --- | --- | --- | --- | --- |
| M | 66 | 32.9 | yes | yes | no | no | 12.4 | 275 | yes | 4 | 3 | 1 | 2 | high risk |
| F | 64 | 35.0 | yes | yes | yes | no | 55 | 0 | no |  |  |  |  | high risk |
| F | 59 | 45.6 | no | no | no | no | 25.3 | 326 | no |  |  |  |  | high risk |
| M | 70 | 34.9 | no | no | no | no | 14.1 | 314 | yes | 3 | 3 | 1 | 1 | Interm. risk |
| F | 58 | 38.8 | yes | yes | no | no | 20.1 | 400 | yes | 2 | 3 | 0 | 1 | Interm. risk |
| F | 54 | 43.9 | yes | no | no | no | 31.5 | 391 | yes | 3 | 3 | 1 | 1 | Interm. risk |
| M | 34 | 39.4 | no | no | no | no | 6 | 275 | no |  |  |  |  | low risk |
| F | 68 | 36.6 | no | no | no | no | 6.1 | 327 | no |  |  |  |  | low risk |
| M | 33 | 24.2 | no | no | no | no | 4.6 | 259 | no |  |  |  |  | low risk |
| M | 68 | 36.0 | yes | yes | no | yes | 7.3 | 352 | no |  |  |  |  | low risk |
| M | 49 | 29.0 | yes | yes | no | yes | 8.4 | 367 | no |  |  |  |  | low risk |
| M | 43 | 38.5 | no | no | no | no | 5.8 | 360 | no |  |  |  |  | low risk |
| M | 29 | 30.1 | no | no | no | no | 4 | 281 | no |  |  |  |  | low risk |
| F | 66 | 31.5 | yes | yes | no | no | 7.8 | 338 | no |  |  |  |  | low risk |
| M | 75 | 33.9 | no | no | no | no | 6.7 | 345 | no |  |  |  |  | low risk |
| F | 56 | 28.9 | no | no | no | no | 8.4 | 275 | yes | 2 | 3 | 0 | 1 | low risk |
| M | 31 | 39.1 | no | no | no | no | 4.8 | 319 | no |  |  |  |  | low risk |
| **Abbreviations:** **M:** male, **F:** Female, **DM:** Type 2 Diabetes Mellitus, **LS**: Liver stiffness, **CAP**: controlled attenuation parameter, **F:** Fibrosis stage, **S**: Steatosis grade, **LI:** Lobular inflammation, **B**: hepatocyte ballooning | | | | | | | | | | | | | | |

**Supplementary Table 2** Serum biochemistry data at baseline

|  | Healthy | Low risk (MASLD) | High risk (MASLD) |
| --- | --- | --- | --- |
| Liver function |  |  |  |
| ALT (IU/L) | 11 ± 5 | 33 ± 17^**^ | 26 ± 7^***^ |
| AST (IU/L) | 16 ± 5 | 28 ± 9^**^ | 36 ± 14^**^ |
| Total Bilirubin (μmol/L) | 8.6 ± 2.8 | 9.6 ± 8.3 | 20.7 ± 17.7 |
| GGT (IU/L) | 12 ± 2 | 72 ± 74^*^ | 131 ± 157^*^ |
| CRP (mg/L) | 0.84 ± 0.86 | 3.9 ± 4.3^*^ | 2.7 ± 1.8^*^ |
| Renal function |  |  |  |
| Urea (mmol/L) | 4.7 ± 1.4 | 5.8 ± 2.4 | 5.6 ± 1.4 |
| Creatinine (μmol/L) | 74 ± 10 | 81 ± 21 | 63 ± 14 |
| Serum biochemistry at baseline between healthy, low risk (MASLD) and high risk (MASLD). Alanine aminotransferase (ALT), aspartate aminotransferase (AST), gamma-glutamyl transferase (GGT), C-reactive protein (CRP). Data presented as mean ± standard deviation. Unpaired t-test and statical significance is shown between the MASLD groups compared to the healthy volunteers as *p<0.05, **p<0.01, ***p<0.001, ****p<0.0001. No significance was found between the two MASLD groups. | | | |

**Supplementary Table 3** Baseline cardiac data

|  | Healthy | Low risk MASLD | High risk MASLD |
| --- | --- | --- | --- |
| Cardiac function |  |  |  |
| LV Stroke Volume (mL) | 100 ± 32 | 106 ± 21 | 105 ± 34 |
| LV Ejection Fraction (%) | 62 ± 4 | 63 ± 5 | 67 ± 4^*^ |
| RV Stroke Volume (mL) | 86 ± 28 | 100 ± 21 | 94 ±35 |
| RV Ejection Fraction (%) | 59 ± 4 | 62 ± 7 | 68 ± 7^**^ |
| Myocardial mass (g) | 110 ± 42 | 126 ± 27 | 108 ± 20 |
| Heart Rate (bpm) | 53 ± 9 | 67 ± 13^*^ | 78 ± 21^**^ |
| Cardiac Index (L/min/m^2^) | 2.7 ± 0.5 | 3.2 ± 0.7 | 3.7 ± 1.1^*^ |
| Cardiac Metabolite (Plasma) |  |  |  |
| NT-proBNP (pg/mL) | - | 75 ± 83 | 73 ± 37 |
| Cardiac MRS |  |  |  |
| Allylic (%) | 0.18 ± 0.12 | 0.35 ± 0.19^*^ | 0.55 ± 0.60 |
| Methylene (%) | 0.43 ± 0.13 | 0.97 ± 0.49^**^ | 1.50 ± 1.59 |
| Methyl (%) | 0.67 ± 0.27 | 0.21 ± 0.21^***^ | 0.40 ± 0.45 |
| PDFF (%) | 1.37 ± 0.50 | 1.66 ± 0.80 | 2.63 ± 2.1 |
| Cardiac evaluation using MRI, MRS, and blood sample analysis. Healthy volunteers had no history of MASLD or other disease. Low risk (MASLD) is clinical evaluation of participants that have low risk of developing liver complications. High risk (MASLD) is clinical evaluation of participants that have high risk of developing liver complications. Left ventricle (LV), right ventricle (RV), brain natriuretic peptide (BNP). Allylic, methylene, and methyl peaks were calculated as a percentage of the lipid peak (f) plus the unsuppressed water peak (w) (f/(f+w)), proton density fat fraction (PDFF) as evaluated on MRI. Data presented as mean ± standard deviation. Unpaired t-test and statical significance is shown between MASLD and healthy volunteers as *p<0.05, **p<0.01, ***p<0.001, ****p<0.0001. | | | |

**Supplementary Table 4** Liver MRI results at baseline

|  | Healthy | Low risk (MASLD) | High risk (MASLD) |
| --- | --- | --- | --- |
| T_1_ (ms) | 763 ± 73 | 848 ± 105^*^ | 928 ± 178^*^ |
| T_2_^*^ (ms) | 17.6 ± 4.6 | 16.1 ± 2.9 | 16.2 ± 2.6 |
| Data presented as mean ± standard deviation. Unpaired t-test and statical significance is shown between the MASLD groups and healthy volunteers as *p < 0.05. | | | |

**Supplementary Table 5** Two-way ANOVA analysis of the effects of time after L-carnitine injection and participant severity group on the liver as evaluated by magnetic resonance imaging and spectroscopy

|  | Group | Timepoint | | 2-way ANOVA | | |
| --- | --- | --- | --- | --- | --- | --- |
|  |  | **T0** | **T3** | **Time after L-carnitine** | **Group** | **Interaction** |
| Hepatic MRI | | | | | | |
| T_1_ (ms) | HV  l-MASLD  h-MASLD | 763±73  848±105  928±178 | 743±58  857±114  929±175 | 0.67 | 0.018 | 0.20 |
| T_2_^*^ (ms) | HV  l-MASLD  h-MASLD | 17.0±4.7  16.1±2.9  16.2±2.6 | 14.8±4.9  16.1±2.9  15.6±2.0 | 0.27 | 0.98 | 0.47 |
| Hepatic MRS | | | | | | |
| Allylic (%) | HV  l-MASLD  h-MASLD | 0.62±0.38  3.12±1.61  3.26±2.81 | 0.63±0.31  3.01±1.51  4.29±5.91 | 0.61 | 0.0013 | 0.75 |
| Methylene (%) | HV  l-MASLD  h-MASLD | 1.78±2.65  15.86±9.05  15.00±8.92 | 1.77±2.29  15.81±8.77  17.88±18.18 | 0.63 | 0.0003 | 0.81 |
| Methyl (%) | HV  l-MASLD  h-MASLD | 0.29±0.22  1.89±1.16  2.22±1.35 | 0.43±0.76  2.12±1.26  2.84±3.74 | 0.32 | 0.0055 | 0.84 |
| PDFF | HV  l-MASLD  h-MASLD | 2.7±2.0  12.8±7.9  8.9±3.3 | 2.7±2.2  12.7±8.1  9.1±4.0 | 0.89 | 0.0015 | 0.89 |
| Acetylcarnitine | HV  l-MASLD  h-MASLD | 0.059±0.039  0.719±1.417  0.646±0.620 | 0.231±0.440  2.396±3.405  0.502±0.467 | 0.22 | 0.026 | 0.14 |
| Two-way ANOVA analysis between hepatic function evaluated with MRI measured at baseline (T0), and 1 hour (T1), 2 hours (T2), and 3 hours (T3) after L-carnitine supplementation in the three groups (healthy volunteers, low risk MASLD, high risk MASLD). Data presented as mean ± standard deviation. MRI of voxels in the posterior and anterior part of the liver were evaluated. An iron-corrected shMOLLI T_1_ map was used. A two-way ANOVA, where time represents the T0, T1, T2 and T3 timepoints. group represents whether it is healthy volunteers, low risk (MASLD) or high risk (MASLD), and subject represent the individual subjects. Statistical significance is considered for p<0.05. Healthy volunteers (HV), low-risk MASLD (L-MASLD), high-risk MASLD (h-MASLD). | | | | | | |

**Supplementary Table 6** Two-way ANOVA analysis of the effects time after of L-carnitine injection and participant group on cardiac MRI and MRS

| Parameter | Group | Time-points | | 2-way ANOVA | | |
| --- | --- | --- | --- | --- | --- | --- |
|  |  | **T0** | **T3** | **Time after L-carnitine** | **Group** | **Interaction** |
| Cardiac function | | | | | | |
| LV Stroke Volume (mL) | HV  l-MASLD  h-MASLD | 99.4±30.1  106.0±20.7  104.5±34.4 | 101.8±38.7  105.4±22.4  105.1±40.2 | 0.85 | 0.93 | 0.99 |
| LV Ejection Fraction (%) | HV  l-MASLD  h-MASLD | 62.5±3.8  63.0±4.9  66.5±4.2 | 63.5±2.9  63.2±4.9  67.2±5.5 | 0.62 | 0.039 | 0.96 |
| RV Stroke Volume (mL) | HV  l-MASLD  h-MASLD | 87.4±26.8  100.1±21.2  100.8±35.3 | 90.6±26.5  101.8±21.5  103.2±44.7 | 0.76 | 0.31 | 0.99 |
| RV Ejection Fraction (%) | HV  l-MASLD  h-MASLD | 54.0±13.2  61.8±7.5  68.5±7.1 | 53.8±6.8  62.0±5.5  68.0±9.1 | 0.95 | 0.0001 | 0.99 |
| Myocardial mass (g) | HV  l-MASLD  h-MASLD | 109.6±39.5  126.3±27.4  108.3±20.0 | 111±30.1  126.5±25.5  117.5±22.9 | 0.66 | 0.18 | 0.91 |
| Cardiac Index (L/min/m^2^) | HV  l-MASLD  h-MASLD | 2.7±0.5  3.4±0.6  3.4±1.2 | 2.8±0.4  3.4±0.8  3.4±1.4 | 0.95 | 0.014 | 0.99 |
| Cardiac metabolite (serum) | | | | | | |
| NT-pro-BNP (pg/mL) | HV  l-MASLD  h-MASLD | -  75.5±82.5  72.5±36.8 | -  84.3±101.4  78.3±51.0 | 0.79 | 0.0008 | 0.98 |
| Cardiac MRS | | | | | | |
| Allylic (%) | HV  l-MASLD  h-MASLD | 0.17±0.12  0.35±0.19  0.55±0.60 | 0.15±0.10  0.32±0.22  0.44±0.38 | 0.54 | 0.0037 | 0.90 |
| Methylene (%) | HV  l-MASLD  h-MASLD | 0.43±0.13  0.97±0.49  1.50±1.59 | 0.36±0.09  1.15±0.65  1.04±0.92 | 0.47 | 0.010 | 0.34 |
| Methyl (%) | HV  l-MASLD  h-MASLD | 0.70±0.27  0.21±0.21  0.40±0.45 | 0.69±0.28  0.18±0.14  0.13±0.25 | 0.12 | <0.0001 | 0.23 |
| PDFF (%) | HV  l-MASLD  h-MASLD | 1.39±0.47  1.66±0.80  2.64±2.07 | 1.24±0.43  1.79±0.86  1.69±1.54 | 0.23 | 0.13 | 0.29 |
| Choline (%) | HV  l-MASLD  h-MASLD | 0.32±0.25  0.30±0.25  0.51±0.51 | 0.26±0.27  0.38±0.66  0.14±0.13 | 0.20 | 0.036 | 0.49 |
| Creatine (%) | HV  l-MASLD  h-MASLD | 0.27±0.20  0.17±0.16  0.19±0.12 | 0.29±0.31  0.24±0.39  0.15±0.12 | 0.46 | 0.76 | 0.78 |
| Two-way ANOVA analysis between Cardiac evaluation using MRI, MRS and blood sample analysis measured at baseline (T0), and 1 hour (T1), 2 hours (T2), and 3 hours (T3) after L-carnitine supplementation in the three groups (healthy volunteers, low risk MASLD, high risk MASLD). Data presented as mean ± standard deviation. Left ventricle (LV), right ventricle (RV), brain natriuretic peptide (BNP). Allylic, methylene and methyl peak were calculated as a percentage of the lipid peak (f) plus unsuppressed water (w) (f/(f+w)), proton density fat fraction (PDFF) as evaluated on MRI in the cardiac septum. Healthy volunteers (HV), low-risk MASLD (L-MASLD), high-risk MASLD (h-MASLD). | | | | | | |

**Supplementary Table 7** Two-way ANOVA analysis for differences in serum metabolites over the time course of the experiment and by participant group

| Metabolites | Group | Time points | | | | 2-way ANOVA | | |
| --- | --- | --- | --- | --- | --- | --- | --- | --- |
|  |  | **T0** | **T1** | **T2** | **T3** | **Time after L-carnitine** | **Group** | **Interaction** |
| NEFA | HV  l-MASLD  h-MASLD | 0.48±0.13  0.52±0.23  0.66±0.26 | 0.38±0.14  0.60±0.26  0.72±0.29 | 0.44±0.15  0.62±0.32  0.77±0.31 | 0.39±0.15  0.60±0.26  0.62±0.33 | 0.39 | 0.032 | 0.66 |
| 3-OHB | HV  l-MASLD  h-MASLD | 0.11±0.01  0.11±0.01  0.11±0.02 | 0.12±0.03  0.11±0.02  0.14±0.06 | 0.13±0.05  0.11±0.02  0.15±0.07 | 0.14±0.06  0.11±0.02  0.17±0.08 | 0.006 | 0.19 | 0.11 |
| Cholesterol | HV  l-MASLD  h-MASLD | 4.61±0.86  5.14±1.38  3.83±0.86 | - | - | 4.78±1.13  5.19±1.42  3.84±0.94 | 0.15 | 0.10 | 0.46 |
| TAG | HV  l-MASLD  h-MASLD | 0.89±0.36  2.83±2.33  1.70±1.18 | 0.78±0.27  2.30±1.82  1.53±0.88 | 0.74±0.23  2.28±1.67  1.56±0.84 | 0.78±0.24  2.32±1.67  1.64±0.83 | 0.031 | 0.035 | 0.13 |
| HDL | HV  l-MASLD  h-MASLD | 1.39±0.20  2.39±2.47  2.67±1.19 | - | - | 1.47±0.3  1.97±1.75  1.64±0.83 | 0.36 | 0.51 | 0.21 |
| ALT | HV  l-MASLD  h-MASLD | 10.9±5.4  32.7±17.1  25.7±6.9 | - | - | 11.5±5.2  32.3±16.5  25.1±6.9 | 0.75 | 0.0023 | 0.49 |
| AST | HV  l-MASLD  h-MASLD | 16.4±4.6  28.0±8.8  36.3±13.6 | - | - | 18.6±4.8  29.5±9.9  38.0±14.9 | 0.0075 | 0.0019 | 0.86 |
| AST/ALT ratio | HV  l-MASLD  h-MASLD | 1.74±0.57  0.97±0.28  1.43±0.46 | - | - | 1.84±0.55  1.03±0.31  1.50±0.38 | 0.028 | 0.0012 | 0.86 |
| Urea | HV  l-MASLD  h-MASLD | 4.66±1.14  5.79±2.24  5.58±1.42 | - | - | 4.23±0.93  5.27±2.17  5.14±1.40 | 0.0002 | 0.35 | 0.90 |
| Creatinine | HV  l-MASLD  h-MASLD | 73.7±9.9  81.4±20.7  62.8±14.3 | - | - | 71±10.2  79.6±21.6  62.0±15.8 | 0.016 | 0.12 | 0.60 |
| CRP | HV  l-MASLD  h-MASLD | 0.84±0.86  3.9±4.3  2.7±1.8 | - | - | 0.8±1.0  3.8±4.2  2.7±2.1 | 0.79 | 0.095 | 0.60 |
| Total bilirubin | HV  l-MASLD  h-MASLD | 8.6±2.8  9.6±8.3  20.7±17.7 | - | - | 10.9±2.5  10.7±7.6  24.3±21.0 | 0.0001 | 0.056 | 0.15 |
| Insulin | HV  l-MASLD  h-MASLD | 47.0±17.4  118.7±35.8  367.1±348.4 | 33.6±13.9  112.7±55.1  360.5±365.9 | 31.0±13.0  96.6±41.6  347.0±394.1 | 21.9±11.0  72.1±26.3  272.6±377.9 | <0.0001 | 0.0091 | 0.027 |
| Glucose | HV  l-MASLD  h-MASLD | 4.9±0.4  6.8±2.4  8.1±3.2 | 4.7±0.4  6.7±2.2  7.9±3.2 | 4.7±0.4  6.5±1.9  7.6±3.0 | 4.8±0.3  6.2±1.6  7.1±2.7 | 0.0001 | 0.026 | 0.013 |
| GGT | HV  l-MASLD  h-MASLD | 12.4±1.8  71.9±74.1  130.9±157.2 | - | - | 12.7±1.8  71.7±73.8  131.1±156.7 | 0.92 | 0.054 | 0.97 |
| Lactate | HV  l-MASLD  h-MASLD | 1.6±1.2  1.5±0.4  1.8±0.7 | 1.1±0.5  1.4±0.3  1.5±0.4 | 1.0±0.3  1.2±0.3  1.4±0.5 | 1.1±0.2  1.5±0.5  2.2±1.0 | 0.026 | 0.047 | 0.24 |
| HOMA-IR | HV  l-MASLD  h-MASLD | 1.50±0.65  5.06±2.05  24.63±35.90 | 1.04±0.50  4.85±2.74  24.11±36.89 | 0.95±0.46  3.96±1.91  22.86±37.38 | 0.68±0.39  2.82±1.14  17.52±31.76 | <0.0001 | 0.055 | <0.0001 |
| ApoB | HV  l-MASLD  h-MASLD | 0.85±0.24  1.09±0.29  0.77±0.16 | - | - | 0.89±0.28  1.11±0.29  0.79±0.17 | 0.014 | 0.041 | 0.47 |
| Two-way ANOVA analysis between the serum metabolites measured at baseline (T0), and 1 hour (T1), 2 hours (T2), and 3 hours (T3) after L-carnitine supplementation in the three groups (healthy volunteers, low risk MASLD, high risk MASLD). Data presented as mean ± standard deviation non-esterified fatty acids (NEFA), β-hydroxybutyrate (3-OHB), triglycerides (TAG), high-density lipoprotein (HDL), alanine aminotransferase (ALT), aspartate aminotransferase (AST), C-reactive protein (CRP), gamma-glutamyl transferase (GGT), homeostatic model assessment insulin resistance (HOMA-IR), apolipoprotein-b (APOB). Healthy volunteers (HV), low-risk MASLD (L-MASLD), high-risk MASLD (h-MASLD). Some of the metabolites were not measured at T1 and T2 because they were anticipated not to change. | | | | | | | | |

**Supplementary Table 8** Two-way ANOVA analysis on carnitine species measured from serum samples using tandem mass spectrometry

| Carnitine species | Group | Time-point | | | | 2-way ANOVA | | |
| --- | --- | --- | --- | --- | --- | --- | --- | --- |
|  |  | **T0** | **T1** | **T2** | **T3** | **Time after L-carnitine** | **Group** | **Interaction** |
| C0 | HV  l-MASLD  h-MASLD | 35.8±8.6  41.2±11.7  41.7±3.9 | 857.8±122.0  1111.8±433.6  1137.6±162.6 | 432.9±75.7  564.3±94.3  572.2±87.3 | 250.7±65.8  390.9±139.0  356.5±37.9 | <0.0001 | 0.015 | 0.23 |
| C2 | HV  l-MASLD  h-MASLD | 15.3±2.8  15.4±5.6  18.8±7.5 | 28.2±10.4  24.2±7.7  32.1±7.0 | 32.1±12.9  20.3±12.0  38.0±6.8 | 40.7±12.2  35.3±11.9  51.0±9.3 | <0.0001 | 0.16 | 0.05 |
| C3 | HV  l-MASLD  h-MASLD | 0.34±0.15  0.37±0.18  0.41±0.13 | 0.69±0.21  0.79±0.30  0.81±0.29 | 0.94±0.38  1.02±0.38  1.06±0.28 | 1.05±0.54  1.13±0.43  1.21±0.39 | <0.0001 | 0.72 | 0.99 |
| C4 | HV  l-MASLD  h-MASLD | 0.17±0.13  0.22±0.16  0.16±0.07 | 0.25±0.14  0.39±0.24  0.30±0.17 | 0.35±0.20  0.51±0.31  0.43±0.20 | 0.32±0.15  0.49±0.27  0.52±0.25 | <0.0001 | 0.36 | 0.022 |
| C5:1 | HV  l-MASLD  h-MASLD | 0.012±0.007  0.014±0.008  0.013±0.005 | 0.010±0.006  0.014±0.008  0.008±0.003 | 0.006±0.004  0.014±0.010  0.013±0.003 | 0.013±0.008  0.008±0.007  0.011±0.006 | 0.46 | 0.47 | 0.02 |
| C5 | HV  l-MASLD  h-MASLD | 0.083±0.047  0.148±0.134  0.102±0.050 | 0.066±0.044  0.180±0.146  0.118±0.045 | 0.079±0.047  0.190±0.135  0.128±0.068 | 0.072±0.025  0.192±0.118  0.160±0.068 | 0.010 | 0.065 | 0.031 |
| C4-OH | HV  l-MASLD  h-MASLD | 0.006±0.005  0.018±0.016  0.035±0.028 | 0.017±0.019  0.035±0.029  0.099±0.075 | 0.026±0.022  0.050±0.038  0.130±0.112 | 0.033±0.031  0.063±0.047  0.254±0.202 | <0.0001 | 0.0016 | <0.0001 |
| C6 | HV  l-MASLD  h-MASLD | 0.028±0.015  0.102±0.196  0.038±0.013 | 0.043±0.020  0.170±0.309  0.102±0.046 | 0.054±0.019  0.243±0.421  0.129±0.042 | 0.053±0.026  0.218±0.340  0.177±0.080 | 0.0021 | 0.35 | 0.084 |
| C5-OH | HV  l-MASLD  h-MASLD | 0.031±0.013  0.039±0.016  0.031±0.012 | 0.020±0.008  0.039±0.013  0.037±0.015 | 0.019±0.007  0.035±0.016  0.031±0.009 | 0.032±0.010  0.044±0.022  0.041±0.023 | 0.01 | 0.046 | 0.406 |
| C8 | HV  l-MASLD  h-MASLD | 0.132±0.129  0.335±0.694  0.133±0.072 | 0.138±0.131  0.293±0.569  0.102±0.033 | 0.120±0.115  0.397±0.782  0.143±0.044 | 0.130±0.109  0.364±0.594  0.193±0.056 | 0.11 | 0.46 | 0.14 |
| C3DC/C8-OH | HV  l-MASLD  h-MASLD | 0.027±0.015  0.033±0.021  0.053±0.018 | 0.041±0036  0.038±0.027  0.052±0.020 | 0.031±0.026  0.044±0.034  0.050±0.021 | 0.027±0.023  0.042±0.024  0.069±0.031 | 0.12 | 0.16 | 0.02 |
| C10:1 | HV  l-MASLD  h-MASLD | 0.092±0.095  0.157±.0.183  0.141±0.064 | 0.083±0.047  0.118±0.098  0.123±0.033 | 0.088±0.070  0.156±0.141  0.153±0.048 | 0.077±0.041  0.170±0.111  0.198±0.068 | 0.05 | 0.24 | 0.20 |
| C10 | HV  l-MASLD  h-MASLD | 0.259±0.188  0.596±1.090  0.304±0.117 | 0.226±0.151  0.507±0.974  0.274±0.081 | 0.196±0.110  0.655±1.208  0.274±0.081 | 0.210±0.144  0.598±0.873  0.320±0.066 | 0.14 | 0.45 | 0.32 |
| C4DC | HV  l-MASLD  h-MASLD | 0.022±0.010  0.038±0.031  0.039±0.013 | 0.016±0.008  0.038±0.026  0.026±0.016 | 0.014±0.005  0.039±0.025  0.033±0.007 | 0.016±0.006  0.038±0.017  0047±0.020 | 0.16 | 0.011 | 0.24 |
| C5DC/C10-OH | HV  l-MASLD  h-MASLD | 0.063±0.032  0.084±0.076  0.073±0.020 | 0.059±0.025  0.091±0.075  0.048±0.012 | 0.053±0.029  0.090±0.077  0.083±0.026 | 0.055±0.020  0.096±0.066  0.088±0.039 | 0.10 | 0.36 | 0.017 |
| C12:1 | HV  l-MASLD  h-MASLD | 0.052±0.065  0.081±0.085  0.071±0.019 | 0.027±0.013  0.064±0.071  0.088±0.041 | 0.034±0.013  0.099±0.099  0.128±0.076 | 0.037±0.017  0.096±0.079  0.185±0.121 | 0.0006 | 0.065 | <0.0001 |
| C12 | HV  l-MASLD  h-MASLD | 0.044±0.047  0.082±0.125  0.064±0.028 | 0.031±0.020  0.071±0.113  0.054±0.018 | 0.032±0.020  0.090±0.149  0.055±0.014 | 0.027±0.019  0.104±0.166  0.081±0.047 | 0.094 | 0.45 | 0.14 |
| C6DC | HV  l-MASLD  h-MASLD | 0.006±0.007  0.023±0.034  0.016±0.010 | 0.005±0.004  0.019±0.033  0.012±0.009 | 0.004±0.005  0.020±0.029  0.018±0.013 | 0.006±0.005  0.023±0.037  0.013±0.007 | 0.23 | 0.30 | 0.55 |
| C14:1 | HV  l-MASLD  h-MASLD | 0.056±0.059  0.090±0.139  0.088±0.035 | 0.038±0.027  0.089±0.137  0.087±0.071 | 0.041±0.024  0.117±0.164  0.123±0.088 | 0.048±0.030  0.132±0.150  0.190±0.159 | 0.0004 | 0.32 | 0.0013 |
| C14 | HV  l-MASLD  h-MASLD | 0.009±0.007  0.025±0.039  0.021±0.010 | 0.008±0004  0.025±0.032  0.022±0.010 | 0.011±0.002  0.033±0.053  0.032±0.024 | 0.011±0.007  0.041±0.048  0.038±0.024 | 0.001 | 0.25 | 0.20 |
| C14:1-OH | HV  l-MASLD  h-MASLD | 0.004±0.004  0.035±0.029  0.074±0.051 | 0.004±0.003  0.040±0.033  0.064±0.030 | 0.002±0.003  0.041±0.021  0.060±0.039 | 0.005±0.006  0.041±0.023  0.060±0.048 | 0.87 | <0.0001 | 0.75 |
| C14:OH | HV  l-MASLD  h-MASLD | 0.0006±0.002  0.003±0.003  0.005±0.004 | 0.0006±0.002  0.003±0.003  0.008±0.004 | 0.0017±0.003  0.006±0.004  0.007±0.005 | 0.0011±0.002  0.004±0.005  0.013±0.014 | 0.036 | 0.035 | 0.049 |
| C16:1 | HV  l-MASLD  h-MASLD | 0.009±0.007  0.020±0.035  0.021±0.011 | 0.007±0.005  0.021±0.036  0.029±0.020 | 0.009±0.007  0.020±0.049  0.036±0.026 | 0.016±0.007  0.033±0.050  0.054±0.040 | <0.0001 | 0.27 | 0.030 |
| C16 | HV  l-MASLD  h-MASLD | 0.058±0.015  0.072±0.034  0.088±0.019 | 0.058±0.019  0.084±0.039  0.093±0.032 | 0.060±0.018  0.090±0.044  0.093±0.027 | 0.074±0.027  0.101±0.049  0.137±0.044 | <0.0001 | 0.059 | 0.011 |
| C16:1-OH | HV  l-MASLD  h-MASLD | 0.0000±0.000  0.0005±0.002  0.0040±0.004 | 0.0020±0.003  0.0014±0.002  0.0050±0.008 | 0.0010±0.002  0.0064±0.006  0.0030±0.004 | 0.0040±0.004  0.0036±0.004  0.0130±0.009 | <0.0001 | 0.030 | 0.001 |
| C16-OH | HV  l-MASLD  h-MASLD | 0.0000±0.000  0.0009±0003  0.0030±0.004 | 0.0020±0.003  0.0014±0.003  0.0100±0.002 | 0.000±0.000  0.0009±0.003  0.0030±0.003 | 0.001±0.003  0.0032±0.004  0.006±0.004 | 0.024 | 0.039 | 0.26 |
| C18:1 | HV  l-MASLD  h-MASLD | 0.097±0.039  0.092±0.052  0.110±0.041 | 0.094±0.034  0.107±0.058  0.132±0.057 | 0.089±0.027  0.116±0.042  0.153±0.075 | 0.121±0.059  0.140±0.061  0.207±0.110 | <0.0001 | 0.18 | 0.012 |
| C18 | HV  l-MASLD  h-MASLD | 0.024±0.022  0.018±0.011  0.021±0.006 | 0.027±0.024  0.020±0.009  0.020±0.008 | 0.027±0.025  0.020±0.010  0.017±0.011 | 0.027±0.015  0.024±0.011  0.026±0.011 | 0.13 | 0.62 | 0.73 |
| C18:1-OH | HV  l-MASLD  h-MASLD | 0.0000±0.000  0.0009±0.002  0.0030±0.003 | 0.0020±0.003  0.0018±0.004  0.0030±0.004 | 0.0000±0.000  0.0180±0.004  0.0060±0.005 | 0.0010±0.002  0.0018±0.003  0.0080±0.009 | 0.14 | 0.007 | 0.24 |
| C18-OH | HV  l-MASLD  h-MASLD | 0.0000±0.000  0.0000±0.000  0.0008±0.002 | 0.001±0.002  0.0000±0.002  0.0008±0.002 | 0.000±0.000  0.0000±0.002  0.0008±0.002 | 0.001±0.002  0.002±0.003  0.0017±0.003 | 0.039 | 0.97 | 0.94 |
| Summary |  |  |  |  |  |  |  |  |
| Short-chain carnitines | HV  l-MASLD  h-MASLD | 0.609±0.248  0.770±0.427  0.717±0.253 | 1.030±0.334  1.401±0.565  1.339±0.454 | 1.402±0.520  1.778±0.698  1.763±0.511 | 1.489±0.576  1.883±0.781  2.158±0.685 | <0.0001 | 0.27 | 0.16 |
| Medium-chain carnitines | HV  l-MASLD  h-MASLD | 0.672±0.460  1.464±2.343  0.878±0.275 | 0.616±0.319  1.356±2.105  0.770±0.146 | 0.574±0.271  1.756±2.723  1.023±0.269 | 0.608±0.267  1.665±2.056  1.319±0.424 | 0.010 | 0.40 | 0.025 |
| Long-chain carnitines | HV  l-MASLD  h-MASLD | 0.360±0.230  0.545±0.528  0.588±0.165 | 0.305±0.113  0.543±0.483  0.617±0.245 | 0.312±0.101  0.667±0.603  0.733±0.358 | 0.379±0.159  0.748±0.603  1.033±0.612 | <0.0001 | 0.15 | 0.0017 |
| Total carnitine species | HV  l-MASLD  h-MASLD | 52.64±9.71  59.2±17.8  62.6±10.6 | 887.9±122.2  1139.2±437.5  1172.3±166.3 | 467.3±74.5  598.7±104.9  613.6±86.0 | 293.8±67.4  430.4±141.3  411.8±41.7 | <0.0001 | 0.014 | 0.24 |
| Two-way ANOVA analysis between serum carnitine species evaluated with mass tandem spectrometry measured at baseline (T0), and 1 hour (T1), 2 hours (T2), and 3 hours (T3) after L-carnitine supplementation in the three groups (healthy volunteers, low risk MASLD, high risk MASLD). Data presented as mean ± standard deviation. Statical significance is considered p < 0.05. Healthy volunteers (HV), low-risk MASLD (L-MASLD), high-risk MASLD (h-MASLD). | | | | | | | | |

# Supplementary Figures


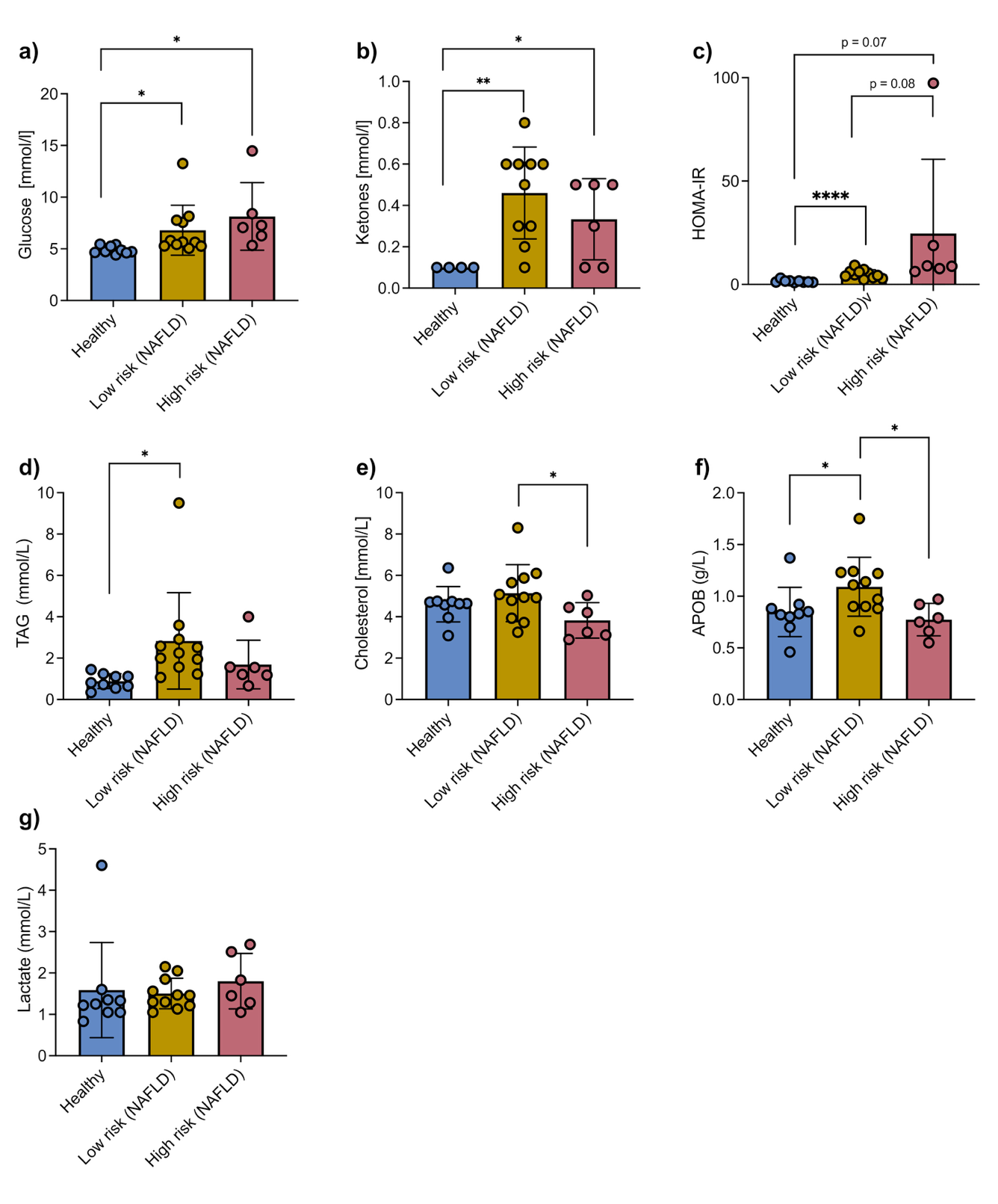


**Supplementary Figure 1** Serum metabolites at baseline (T0). a) serum glucose, b) blood ketones measured directly through a finger prick blood test from a ketone meter, c) homeostatic model assessment for insulin resistance (HOMA-IR) using serum insulin and serum glucose levels, calculated with equation (1), d) triglycerides (TAG), e) cholesterol, f) Apolipoprotein B (APOB), and g) lactate. Unpaired standardized t-test, where *p<0.05, **p<0.01, ***p<0.005, ****p<0.001.

**Supplementary Figure 2** Serum carnitine species at baseline (T0) measured using tandem mass spectrometry: a) free carnitine, C0 (μmol/l), b) acetylcarnitine, C2 (μmol/l), c) C4DC (μmol/l), d) C4-OH (μmol/l), e) C6DC (μmol/l), f) C3DC/C8-OH (μmol/l), g) C14:1 (μmol/l), h) C14 (μmol/l), i) C14:1-OH (μmol/l), j) C14:OH (μmol/l), k) palmitoylcarnitine (C16) (μmol/l), l) C16:1-OH (μmol/l), m) C16-OH (μmol/l). n) C18:1-OH (μmol/l), o) C16:1 (μmol/l), p) short-chain carnitine species, summed[C3-C5] (μmol/l), q) medium-chain carnitine species, summed [C6-C10] (μmol/l), r) long-chain carnitine species, summed [C12-C18] (μmol/l). Unpaired standardized t-test, where *p <0.05, **p<0.01.

**Supplementary Figure 3** Lipid ^1^H MRS acquired from the liver: a) hepatic allylic fat peak normalized to unsuppressed water, b) hepatic methyl fat peak normalized to unsuppressed water, c) hepatic methylene fat peak normalized to unsuppressed water, d) correlation between log-transformed allyl peak amplitude and serum triglycerides, e) correlation between log-transformed methylene peak amplitude and serum triglycerides, f) correlation between log-transformed methylene peak amplitude and serum triglycerides presented as mean ± standard deviation, g) saturation index calculated as (methyl+methylene)/(methyl+methylene+allyl), h) unsaturation index calculated as allyl/(methyl+methylene+allyl)^1^. Standardized unpaired t-test and statistical significance is shown between MASLD and healthy volunteers *p=0.05, ***p<0.001, ****p<0.0001.


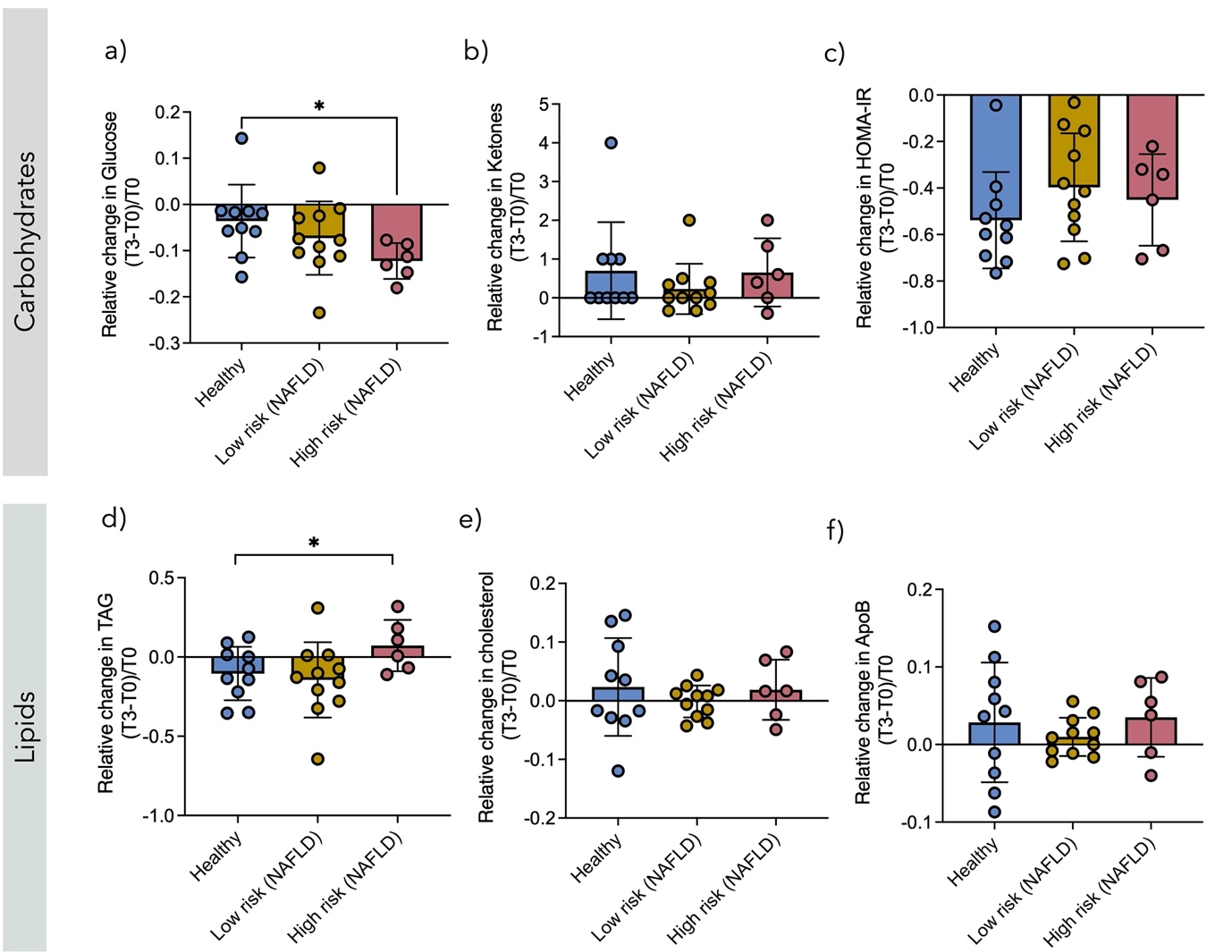


**Supplementary Figure 4** Relative serum metabolite changes between baseline (T0) and at follow-up after 3 hours (T3) after L-carnitine supplementation in healthy volunteers, and patients with low-risk and high-risk MASLD for a) serum glucose, b) blood ketones, c) homeostatic model assessment for insulin resistance (HOMA-IR) using serum insulin and serum glucose levels, d) triglycerides (TAG), e) cholesterol, f) Apolipoprotein B (APOB). Comparisons were done using standardized unpaired t-test (statistical significance indicated as *p<0.05, **p<0.01).
